# Work, Leisure Time Activities, and Mental Health among Family Caregivers of the Elder People in Japan

**DOI:** 10.3390/healthcare9020129

**Published:** 2021-01-28

**Authors:** Tomoko Omiya, Masami Kutsumi, Sakiko Fukui

**Affiliations:** 1 Department of Public Health Nursing, Faculty of Medicine, University of Tsukuba, 1-1-1 Tennnodai, Ibaraki Prefecture, Tsukuba-City 305-8575, Japan; 2 Graduate School of Life and Environmental Sciences, Osaka Prefecture University, 3-7-30 Habikino, Osaka Prefecture, Habikino-City 583-8555, Japan; m-kutsumi@nursing.osakafu-u.ac.jp; 3 Graduate School of Health Care Science, Tokyo Medical and Dental University, 1-5-45 Yushima, Bunkyo-ku, Tokyo 113-8519, Japan; fukuisakiko.chn@tmd.ac.jp

**Keywords:** depression, family caregivers for elderly people, leisure activities, subjective care burden, work

## Abstract

In Japan, there is a high incidence of family members caring for their elderly. To facilitate this, caregivers often quit their jobs, work reduced hours, and forfeit leisure activities. This study examined the relationship between the mental health of the caregivers and the sacrifices and adjustments they make to care for the elderly. A cross-sectional survey was conducted with responses from 171 caregivers. Referencing Pearlin’s stress process model, the relationship among five types of work change, four types of leisure activity quitting, caregivers’ subjective care burden, and depression were analyzed using *t*-test and multiple regression analysis. Caregivers who quit their work or other home activities had significantly more daily living care responsibilities than those who did not. Moreover, caregivers who gave up leisure activities had a greater sense of subjective care burden than those who did not. The experience of giving up peer activities and taking leave of absence from work was significantly associated with increased depressive symptoms. Being a part-timer or financially prosperous was associated with good mental health. To support family caregivers, it is essential to reduce the burden of long-term care and provide financial help and an environment where they interact with their peers, and their moods can be enhanced.

## 1. Introduction

Japan is rapidly moving toward a super-aging society; in 2019, older adults aged 65 and over comprised 28.4% of the population [1]. As families are expected to care for the elderly, a long-term care insurance system was introduced under the concept of “socialization of care” in 2000. The number of persons requiring long-term care certification was 2.18 million in 2000 but increased to 6.44 million in 2018 [2]. The number of insurance care service providers and users is expanding nationwide; however, according to the Basic Survey on National Life, approximately 70% of primary caregivers are family members, either living with care recipients or separately [1]. Thus, the burden on family caregivers is still significant, with more than 50% of primary caregivers reporting being depressed [3]. The burden of caregiving has a significant impact on the caregiver’s mental health, making it difficult for the care recipient to live at home [4], and in some instances resulting in murder and abuse of the care recipient and suicide of family caregivers [5]. The burden of long-term care has a great impact on the mental health of the caregiver, making it difficult for the caregiver to live at home [4], and in some cases leading to the murder and abuse of the caregiver and the suicide of the family caregiver [5]. Moriyama, who conducted a comparative study of family care in the United Kingdom, Finland, and Japan, emphasized the need for Japanese family care providers to maintain quality of life and guarantee their rights as human beings [6]. In some countries in the Nordic and Western Europe regions, the focus is on enhancing institutionalized long-term care facilities, while in Southern and Eastern Europe, it is traditionally common for family care and volunteers to provide long-term care, mainly at home. For example, Germany has long-term care insurance, but the law stipulates that family care is prioritized over businesses, and home care is prioritized over facilities. Due to soaring long-term care costs and a decrease in the working population, the number of long-term care workers is decreasing [7]. Consequently, in countries where institutional care is advancing, family caregivers are more likely to be long-term care providers.

Until now, efforts to recognize the burden on family caregivers have focused on the inter-relationship between the degree of dementia and behavioral problems [8] and families who care for terminally ill patients, such as cancer patients [9]. The burden on caregivers to continue home nursing care for older adults—regardless of the level of care needed—must be reduced [10]. There are also studies examining the burden of long-term care using the psychological stress theory; Pearlin’s elderly care stress process model (SPM) proposes a comprehensive stress process that integrates psychological and sociological perspectives in family caregivers [11]. In the SPM, Pearlin described care recipients’ degree of disability and the state of their cognitive impairment as a primary stressor for family caregivers. Pearlin described the impact of long-term caregiving on the caregiver’s social life as a secondary stressor—for example, the restrictions on leisure time and meetings with friends. The author also presented a model in which the caregivers’ external activity restrictions resulting from providing long-term care are burdensome and lead to depression [11]. When stressors are considered in this way, then the stress diffusion process in older adults’ family caregivers can be viewed in detail from multiple perspectives [11].

Being employed, having a relationship with society, and representing oneself encourage people to improve their quality of life (QOL) and self-esteem. It is also related to human dignity and existence value, such as playing a social role, finding one’s value, and promoting identity formation [12]. However, according to Japan’s Ministry of Internal Affairs and Communications Statistics Bureau’s “Basic Survey on Employment Structure,” about 100,000 people in Japan leave their jobs every year because of family care and nursing obligations [13]. Furthermore, Saito et al. reported that 23.6% of family caregivers left or changed jobs to meet care and nursing responsibilities [14]. Several studies have focused on family caregivers’ work leave, intention to leave (worrying about leaving work for care), and role conflicts [15]. Leaving work to focus solely on long-term care leads to a devotion to long-term care, increasing the mental and physical burden of the caregivers. Simultaneously, quitting work for long-term care means losing a financial base, increasing anxiety and distress [16]. Therefore, it is conceivable that family caregivers will go through various conflicts about leaving their jobs, changing jobs, seeking reassignment, and changing their working routine to part-time employment. However, there have not been many studies that have carefully examined the nursing care situation of people requiring long-term care and the detailed “changes” in working routine styles such as leaving or taking leave.

Similar to working, activities related to QOL and mental health include social activities and leisure activities. Wakui et al. found that family caregivers who engaged in home and social activities experienced lower caregiving burden and depressive symptoms; additionally, the authors reported a positive relationship between participation in social activities and life satisfaction [17]. Similarly, Schüz et al. found that leisure activities increased satisfaction in caregivers of families with dementia [18]. Consequently, a lack of leisure activities could have enormous implications for family caregivers’ mental health, but research is currently minimal.

Previously, the dichotomy between labor and leisure was a mainstream idea [19]. Leisure and work conflicted; leisure was a means of recovering from work. As work and leisure are different or conflicting concepts, the discussion was centered on balancing work and leisure. According to Abe, this dichotomy is no longer valid [20]. Labor is not just a way to earn money, and leisure activities are not just vacations. Instead, work and leisure can both be considered important to an individual’s QOL, as both meet the “need for self-sufficiency” and help the individual develop their abilities [20]. At the same time, as modern people achieve self-actualization through labor, leisure activities are also an important means of being themselves. Given that work and leisure are the two wheels of a car [20], it seems essential to keep both in mind when considering a family caregiver’s mental health and quality of life.

Using Pearlin’s stress model as a theoretical framework, the purpose of this study was to examine and clarify the relationship between caregiver’s mental health, employment, and leisure activities. Specifically, we investigated the severity of the care recipient’s disability and cognitive impairment as a primary stressor to the caregiver; as a secondary stressor, we explored the different caregiving situations that required the continuation or interruption of employment or leisure activities. Additionally, we investigated how these interruptions are related to depression and the burden of long-term care. For work, we considered detailed situations such as changing jobs and short-term leave. For leisure, we considered the content and type of leisure (whether alone or with friends) and suggested specific support for family caregivers.

## 2. Materials and Methods

### 2.1. Participants

To determine the minimum sample size required for this study, we used an α (error probability or significance level) of 0.05, a power level of 0.95, and an effect size (*f*^2^) of 0.3. The suggested minimum sample size was 134 participants. The questionnaire’s return rate was predicted to be about 50%, and the goal was to distribute 300 copies. From January to February 2012, with the cooperation of home visiting nurses from 15 visiting nursing stations in Tokyo and Saitama Prefecture, 288 copies of anonymous, self-administered questionnaires were distributed to families. In total, 171 responses were collected by mail. The response rate was 59.4%.

### 2.2. Caregivers

The caregivers provided data about age, gender, average years of care, current employment status, personal household financial situation, caregiver attributes, and the number of secondary caregivers.

### 2.3. Amount of Care

To measure the amount of care provided by the caregivers, ten items of the Activities of Daily Living (ADL) and seven items of the Instrumental ADL (IADL) checklists proposed by Sugiura et al. were used [21]. ADL items asked about the care of fundamental needs, including food assistance, toileting, dressing and undressing, bathing assistance, transfer from a wheelchair, changing diapers, going up and down stairs, medication, and walking assistance. Moreover, one point was given for every applicable activity (ranging from zero to ten).

IADL items were concerned with social function and included meal preparation, shopping, laundry cleaning, telephone, money management, hospital transfer, and out-of-home assistance. For every activity that applied, one point was given (ranging from zero to seven). The higher the score, the more the amount of care provided.

### 2.4. Cognitive Impairment

Cognitive impairment was assessed using 16 items from the questionnaire created by the Tokyo Metropolitan Institute of Gerontology, which was tested for reliability and validity [21,22]. This questionnaire was developed so that caregivers can easily check the presence or absence of problematic symptoms and behaviors relatively common in older adults with dementia. Sample items included, “He/She says he/she has not eaten just after eating” or “He/She has difficulty remembering his/her age.” Answers were indicated with a “yes” (one point) or “no” (zero points), and total scores ranged from zero to 16. According to the developers’ classification method, a score of 0 meant “no cognitive impairment” and scores from 1–16 meant “with cognitive impairment.” However, in this study, the total score was used as in previous studies [21,22]; higher scores indicated severe cognitive impairment.

### 2.5. Subjective Care Burden

Researchers posited that the burden of long-term care is related to depression [23], and mental health issues such as depression may develop into secondary problems such as abuse [24]. It is important to clarify the factors leading to this from a comprehensive perspective. The caregiving burden was assessed with the question, “How much do you feel burdened by nursing care now?” Answers were recorded on a four-point Likert scale ranging from feeling quite burdensome (four points) to not feeling burdensome (one point) [21].

### 2.6. Caregivers’ Mental Health

In previous studies, significant associations between subjective care burden and depression of caregivers have been reported [23]. In this study, along with the subjective feeling of care burden, we used the Center for Epidemiologic Studies Depression Scale (CES-D), an objective index of depression. CES-D was developed by the United States National Institute of Mental Health to detect depression in the general population [22]. This study evaluated mental health using 11 short-form CES-D items created by Shima et al. and confirmed to be highly reliable and valid [25]. Higher scores reflected a higher degree of depression. Answers were rated using a three-point Likert scale ranging from frequently or most of the time (two points) to hardly ever or never (zero points). The total points were scored by simple addition. Cronbach’s α was 0.845.

### 2.7. Working Situation

For working situations, we referred to “2012 edition of the actual situation of working women, Chapter 2, balancing work and long-term care to continue working without leaving the job” [26] and previous research [16]. We also set question items through discussions among researchers. Caregivers were asked about changes in work caused by family nursing. Experiences like quitting or changing jobs because of caring, taking a long absence from work (taking care leave), reducing work, and changing the employment condition (from regular to part-time work) were assessed. Caregivers were asked to answer two yes/no questions, and one point was given for a “yes” answer.

### 2.8. Leisure Activities

Leisure activities were divided into four activities, based on Wakui et al. [17,27]. In-home activity was defined as a hobby or leisure activity conducted at home: reading books, listening to music, drawing, exercises, horticulture, and handicrafts. Out-of-home personal leisure activity was defined as an activity easily performed by individuals outside their homes: walking, eating out, drinking coffee, and shopping. Out-of-home group activities included activities held outside of the home with friends and companies: hobby associations and family caregiver associations. Out-of-home cultural and entertainment activities included sports, travel, watching movies, and viewing art outside of the home. Participants were asked if they had ever quit any of those activities since they started home care nursing. If they answered “Yes,” they were given one point.

### 2.9. Analysis Method

Regarding each item of work and leisure activities, family caregivers were divided into two groups, one group who answered “yes” to “have experience of quitting or changing for long-term care” and the other group who answered “no”. Using *t*-tests, the difference between the two groups in the amount of ADL and IADL care, the score of cognitive impairment scale, number of secondary caregivers, and caregiver mental health (subjective care burden, CES-D) were analyzed separately for changes in work and leisure activities.

Multiple regression analysis was performed with subjective care burden and CES-D as the dependent variables and the following explanatory variables: attributes and characteristics (gender, age, living direction), the amount of care provided (ADL, IADL, cognitive impairment score, the number of secondary caregivers), based on whether they quit four leisure activities and the presence or absence of each change in work. Each experience of change in work and leisure activities is counted separately. For example, a family caregiver may have multiple experiences, such as giving up an old hobby at home and quitting group activities outside of home. In the multiple regression analysis, each item was inputted according to previous studies [28]. The selection of variables used in the analysis was decided through discussions among researchers about previous research [21,26]. The Statistical Package for Social Sciences (SPSS) version 24.0 was used for the analysis.

### 2.10. Ethical Considerations

The survey was conducted with the approval of the Toho University Faculty of Nursing research ethics review committee (approval number 23025). The participants of the study were given an anonymous survey to complete. They were also given a document stating that participation in the survey was voluntary. The document also explained that there would be no adverse effect, disadvantages, or consequences to those who refused to participate and their family members. In addition to the document, these provisions were also verbally explained to the participants. Additionally, we informed the participants that returning the survey forms would be regarded as consent to participate.

## 3. Results

### 3.1. Attributes and Characteristics of Participants

Table 1 shows the attributes and characteristics of the participants. Nearly 80% of the sample were women. The average age of family caregivers was 63.9 years ± 10.8 years, with those in their 50s and 60s accounting for more than 60% of the sample. The attributes of caregivers were highest for wives (wife caring for husband) and closely followed by daughters (daughter caring for parents). The average length of care was 7.9 ± 8.7 years. At the time of the survey, 56 (32.7%) of the participants were employed, and 115 (67.3%) were unemployed. The average number of secondary caregivers was 1.7 ± 1.1.

Of the caregivers, 26.9% (46) said they had quit their job to provide nursing care, and 72.5% (124 people) said they gave up leisure activities. The average age of those requiring care was 77.5 ± 16.3 years, with 45.6% (78) being men and 54.4% (93) women.

### 3.2. Changes in Work Caused by Nursing Care

The *t*-test results (Table 2) showed that those who quit their jobs to provide nursing care had significantly more ADL caregiving responsibilities than those who had not resigned. Those who reduced their workload provided significantly more IADL care than those who did not. There was no difference in subjective caregiving burden and CES-D score for caregivers who resigned or reduced their workload.

### 3.3. Quitting Leisure Activities Due to Nursing Care

As presented in Table 3, caregivers who quit in-home activities (reading, listening to music, gardening, handicrafts) and outdoor personal leisure activities (walking, eating out, drinking coffee, shopping) had significantly more ADL caregiving responsibilities than those who did not. Regardless of the type of leisure activity, caregivers who gave up leisure activities experienced significantly more caregiving burden. The experience of leaving out-of-home group activities resulted in significantly higher CES-D scores.

### 3.4. Subjective Care and Related Factors

Multiple regression analysis was performed with subjective care burden as the dependent variable to determine factors related to mental health (Table 4). A high amount of ADL care (β = 0.250, *p* = 0.013) and experience of quitting out-of-home activities (β = 0.201, *p* = 0.020) indicated a significant association with higher subjective care burden. 

### 3.5. CES-D and Related Factors

Multiple regression analysis was performed with CES-D as the dependent variable to determine factors related to mental health (Table 5). The experience of stopping group activities outside the house (β = 0.176, *p* = 0.042) and taking care leave (long absence from work) for nursing care (β = 0.217, *p* = 0.023) indicated a significant association with higher CES-D scores. However, a good household economic situation (β = −0.189, *p* = 0.019) and the experience in changing the employment condition (β = −0.258, *p* = 0.023) showed a negative association with the CES-D.

## 4. Discussion

Based on Pearlin’s stress process model, this study examined the long-term subjective care burden and depression of family caregivers. We examined both work and leisure activities as secondary stressors. By simultaneously inputting work and leisure activities in multivariate analysis, we showed that these might have different effects on mental health. Until now, caregivers’ subjective feelings of care burden and mental health, such as depression, have been known to be similar in terms of related factors. However, this study’s multiple regression analysis results (Table 4 and Table 5) suggested that each structure may be different. While it is important to reduce the actual burden of long-term care, factors such as emotional support, interaction with people, and financial support are indispensable.

Previous studies have focused on supporting the balance between care and work, especially in Japan [16,29]. Similarly, as shown in Table 2 and Table 3, a large amount of ADL care was related to quitting work, quitting indoor leisure, and out-of-home personal activities. However, work-related changes such as quitting work and reducing work were not associated with the subjective care burden or deterioration of CES-D. A possible reason for this is the age of the subjects in this study. The average age of the subjects in this study was 63.9 years (SD10.8). The general retirement age in Japan is 65 years, implying that most people have already quit their jobs, meaning that leisure activities may have had strong mental health-related effects. As they have already quit their jobs, these factors need to be considered in generalizing the results.

As shown in Table 3, giving up leisure activities was strongly associated with subjective care burden and CES-D, regardless of the type of leisure activities. The Coleman and Iso–Ahola leisure model of health states that participation in activities such as cultural exchanges, charities, sports, dance, and religious events that are different from the caregiving role result in reduced feelings of care burden [30]. Since the current study is a cross-sectional study, cause and effect cannot be determined. However, this supports the results of our study and shows that it is important to focus on work and leisure activities. It is necessary to understand how caregivers spend their time other than providing long-term care and how they have changed their daily lives.

Table 4 shows a significant positive association between the subjective care burden and the amount of ADL care, and our results indicate that the absolute amount of care is directly related to feelings of burden. These feelings could be significantly resolved by increasing the number of long-term care services, and providing respite care, a traditional means of helping caregivers. The experience of quitting personal activities outdoors was also significantly associated with an increased subjective long-term care burden. It has been suggested that objective interventions such as enhancement of long-term care services and long-term care guidance are supportive for caring for caregivers [31]. However, since the feelings of “too much” and “exhausted” are subjective feelings, it is important to pay attention to caregivers’ subjective aspects and cognitive coping. In other words, in addition to providing objective interventions to reduce the amount of long-term care, it is necessary to provide entertainment support, easing the family caregivers’ subjective burden and facilitating rich, satisfying emotions. Romero-Moreno et al. reported that family caregivers who enjoyed entertainment activities developed a feeling of guilt that they were not attentive enough or failed to fulfill their responsibilities toward care recipients [32]. Strang et al. also reported that female caregivers felt hesitant about performing personal activities or relaxing [33]. This indicates that caregivers need support and leisure activities that affect relaxed emotions. Along with support to reduce the amount of physical care, it is also important to create a mechanism and culture that makes it okay for caregivers to leave caregiving and enjoy activities outside of the home.

Our findings showed that mental health deterioration was significantly associated with abstinence from mood-changing activities with outside peers and long-term care leave. Interaction between peers with the same hobby has the effect of infusing life with purpose and meaning, which is consistent with the results of Wakui et al. [17]. According to Omiya [34], in addition to “changing mood” and “enjoyment of being absorbed in” activities with friends, caregivers may receive encouragement and praise from their peers for providing elderly care, which is a great pleasure for hard-working caregivers. Like self-help groups and long-term care family associations, caring and empathy play a vital role in depression prevention and commitment to long-term care. Caregivers need to deepen their interaction with others as much as possible. Support from visiting nurses and care managers is a catalyst for balancing long-term care and leisure activities, and active advice from healthcare professionals may be important.

Regarding working, CES-D scores were significantly higher for caregivers who took long time care leave. Sugiura et al. reported that short-stay use was associated with worse mental health in male family caregivers [35]. A short stay is a system expected to be used as a respite to maintain caregivers’ physical and mental health. However, it can be interpreted, by Sugiura et al., that the short stay is used because mental health deteriorated, making it difficult to continue nursing care. The study participants also wanted to retire, however, they could not financially afford to quit their jobs, explaining why they chose to take leave from work.

There was a significant negative association between changing working styles and CES-D. In the questionnaire, changing the way of working meant reducing work, such as becoming a part-time worker. The findings suggest that those who balance work and care by changing work styles rather than retiring or taking a leave of absence maintained better mental health. Alternatively, it can be interpreted that even with a reduced salary as a part-time worker, economically good living conditions were retained. The results of this study showed a significant positive association between financial affordability and good mental health. This result implies that financial stability may maintain caregivers’ mental health. Thus, healthcare professionals who support elderly care should carefully assess the family background and introduce necessary services, such as financial assistance and reassembly of services.

A limitation of this study was its cross-sectional design since causality cannot be specified. It is difficult to determine whether caregivers quit their jobs because of the high burden of long-term care or to focus on providing long-term care, which, in turn, made them feel more burdened. Additionally, most of the participants in this study were over 60 years old. Targeting the working generation is necessary to longitudinally investigate how people engaged in work or leisure activities are affected by changes in long-term care conditions. A cohort study of the working population can help understand the impact of work changes. Further, the R-squares of multiple regression analysis for subjective care burden was low; therefore, it is important to revisit the variables.

Additionally, in this study, “people who have never quit their jobs” may have included “people who have not originally worked (housewives, etc.).” As this is a basic study that targeted families who use home nursing in a small area, it was considerably difficult to limit the target population to only those who have experienced working. In Japan, women are still responsible for nursing care and child-rearing, and the reality is that most women in their 60s retired when they got married and became full-time housewives. Larger and more detailed studies are desired, focusing on working people. Nevertheless, in this study, the meaning of having “experience of quitting work”—the impact of having the family caregiver quit “work” or change jobs—seems to have been verified. Our study reported that for adults, the experience of quitting or changing a mentally and economically important “work” could be extremely significant. Further research development aimed at balancing work, leisure activities, and long-term care is desired.

It should also be noted that 78.9% of the subjects were women. Previous studies have shown that gender-specific factors related to stress processes and mental health differ for family care providers [35,36]. Research on male family care providers is also required.

Finally, this study was conducted during 2011–2012. Since then, the social situation has changed, such as nursing care fees have increased. Further aging of the population, increase in households of elderly couples with one child, and the shift to nuclear families in Japan have decreased the number of family caregivers who can be responsible for elderly care. At the same time, this can lead to an increased burden per family caregiver [37].

In Japan, the population is aging at an unprecedented rate. Although long-term care insurance was introduced early, long-term care is financially challenging due to the decrease in the working-age population. The measures Japan will take regarding long-term care and the implications evince worldwide attention [34]. Especially in Asian countries, family care is often the focus for the elderly; maintaining the QOL of family caregivers is extremely important. Given the meaning and purpose of life of long-term care providers and their QOL, it is extremely meaningful to consider work and leisure activities that are more closely related to their QOL.

## 5. Conclusions

In this study, we focused on how changes in work and leisure activities affected the mental health of 171 family caregivers in Japan. Caregivers who gave up their leisure activities were much more burdened with their daily caregiving responsibilities than those who did not, indicating that support is important to alleviate their burden of care. It is likely that family caregivers’ interaction with peers will have a positive impact on their mental health; support for emotional and subjective feelings is just as indispensable as objective support. Laying a financial foundation could also help prevent depression in family caregivers. Quitting work for long-term care may cause depression as well as financial instability. In the future, it is desirable to conduct a prospective study on how long-term care affects the work and leisure activities of family caregivers in their 40 s and 50 s.

## Figures and Tables

**Table 1:** Demographic characteristics of the participants (*n* = 171).

Variables of the Subjects	Items	*n*	%	Mean	SD
Caregiver					
Gender	Male	36	21.1		
	Female	135	78.9		
Average age				63.9	10.8
Average years of care				7.9	8.7
Financial situation	Very poor	16	9.6		
	Poor	38	22.8		
	Average	64	38.3		
	Rich	42	25.1		
	Very rich	8	4.8		
Caregiver attributes	Husband	18	10.7		
	Wife	53	31.5		
	Daughter	51	30.4		
	Son	15	8.9		
	Daughter in law	13	7.7		
	Other	18	10.7		
Number of secondary caregivers (0–9)				1.7	1.1
Experiences related to work and leisure activities					
Quitting work for nursing care (yes)		46	26.9		
Giving up leisure activities for nursing care (yes)		124	72.5		
Mental Health					
Subjective Care Burden (1–4)				3.1	0.7
CES-D ^(a)^ (0–22)				8.0	4.3
Care Recipient					
Age				77.5	16.3
Gender	Male	78	45.6		
	Female	93	54.4		
ADL ^(b)^ care provided	(0–10)			4.4	2.7
IADL ^(c)^ care provided	(0–7)			4.1	2.4
Cognitive impairment	(0–16)			1.7	2.4

(^a^) Epidemiological studies-depression, (^b^) Activities of daily living, (^c^) Instrumental activities of daily living.

**Table 2:** Relationship between changes in work caused by nursing care, nursing care status, and mental health.

	Experience of Quitting Work			Experience of Reducing the Amount of Work			Experience of Changing Jobs			Experience of Taking a Long Leave from Work (Taking Care Leave)			Experience of Changing the Employment Condition		
Variables	Yes *n* = 30	No *n* = 140	*p*-Value	Yes *n* = 26	No *n* = 144	*p*-Value	Yes *n* = 6	No *n* = 165	*p*-Value	Yes *n* = 4	No *n* = 167	*p*-Value	Yes *n* = 12	No *n* = 159	*p*-Value
Care recipient status															
ADL ^(a)^ care provided (0–10)	5.0	4.1	0.045	5.2	4.2	0.082	3.7	4.4	0.531	4.2	4.4	0.932	4.1	4.4	0.721
IADL ^(b)^ care provided (0–7)	4.5	4.0	0.268	5.1	4.0	0.024	4.3	4.1	0.838	3.5	4.2	0.596	3.8	4.2	0.655
Number of secondary caregivers	1.5	1.8	0.185	1.8	1.7	0.616	1.8	1.7	0.774	1.0	1.7	0.189	1.3	1.7	0.131
Cognitive impairment (0–16)	2.3	1.5	0.085	2.7	1.5	0.060	2.0	1.7	0.773	0.5	1.8	0.297	1.9	1.7	0.772
Caregivers’ Mental Health															
Subjective care burden (1–4)	3.2	3.1	0.274	3.1	3.1	0.902	3.0	3.1	0.731	2.5	3.1	0.091	2.9	3.1	0.361
CES-D ^(c)^	8.9	7.7	0.146	7.6	8.1	0.675	6.1	8.1	0.286	12.3	7.9	0.417	6.3	8.1	0.230

(^a^) Activities of daily living, (^b^) Instrumental activities of daily living, (^c^) The center for epidemiologic studies depression scale.

**Table 3:** Relationship between quitting in leisure activities caused by nursing care, nursing care status, and mental health.

	Experience of Quitting In-Home Activities ^(a)^			Experience of Quitting Out-of-Home Personal Activities ^(b)^			Experience of Quitting Out-of-Home Group Activities ^(c)^			Experience of Quitting Cultural and Entertainment Out-of-Home Activities ^(d)^		
Variables	Yes *n* = 30	No *n* = 140	*p*-Value	Yes *n* = 55	No *n* = 116	*p*-Value	Yes *n* = 47	No *n* = 124	*p*-Value	Yes *n* = 63	No *n* = 108	*p*-Value
Care recipient status												
ADL ^(e)^ care provided (0–10)	5.7	4.1	<0.001	5.0	4.1	0.041	4.7	4.2	0.255	4.7	4.2	0.285
IADL ^(f)^ care provided (0–7)	4.7	4.1	0.134	4.3	4.0	0.394	4.6	4.0	0.124	4.3	4.1	0.577
Number of secondary caregivers	1.8	1.6	0.610	1.9	1.6	0.069	1.8	1.7	0.667	1.9	1.6	0.129
Cognitive impairment (0–16)	2.1	1.6	0.336	2.1	1.6	0.165	2.1	1.6	0.214	2.0	1.5	0.197
Caregivers’ Mental Health												
Subjective care burden (1–4)	3.4	3.0	0.007	3.3	2.9	0.004	3.3	3.0	0.020	3.3	3.0	0.025
CES-D ^(g)^	8.1	8.0	0.849	9.1	7.5	0.031	9.5	7.5	0.008	8.9	7.5	0.057

(^a^) In-home activities: reading books, listening to music, drawing, exercises, horticulture, handicrafts. (^b^) Out-of-home personal leisure activities: walking, eating out, drinking coffee, and shopping. (^c^) Out-of-home group activities: neighborhood associations, hobby associations, and family caregiver associations. (^d^) Cultural and entertainment out-of-home activities: sports, travel, watching movies, and watching art. (^e^) Activities of daily living, (^f^) Instrumental activities of daily living, (^g^) The center for epidemiologic studies depression scale.

**Table 4:** Multiple regression analysis results with subjective care burden as the dependent variable (*n* = 171).

Variables	Coefficient β	*p*-Value	95% Cl		
Demographic characteristics					
Gender (female = 1, male = 0)	−0.002	0.983	−0.283	to	0.276
Age	−0.020	0.808	−0.012	to	0.009
Household economic situation (1 = Very poor to 5 = Very rich)	−0.101	0.192	−0.176	to	0.036
Care Recipients’ Situation					
ADL care provided ^(a)^	0.250	0.013	0.014	to	0.116
IADL care provided ^(b)^	−0.080	0.401	−0.079	to	0.032
Cognitive impairment	−0.110	0.191	−0.081	to	0.016
Number of secondary caregivers	−0.081	0.301	−0.154	to	0.048
Experience of caregiver giving up leisure activity (Have quit = 1, Never = 0)					
In-home activities ^(c)^	0.068	0.407	−0.173	to	0.425
Out-of-home personal activities ^(d)^	0.201	0.020	0.049	to	0.559
Out-of-home group activities ^(e)^	0.096	0.245	−0.105	to	0.408
Cultural and entertainment out-of-home activities ^(f)^	0.117	0.174	−0.077	to	0.420
Experience of caregiver changing work situation (Have quit = 1, Never = 0)					
Quitting jobs	0.033	0.675	−0.200	to	0.308
Changing jobs	−0.025	0.783	−0.773	to	0.583
Taking care leave (long absence from work)	−0.167	0.056	−1.797	to	0.022
Changing the employment condition (ex. From full time to part time job)	−0.070	0.468	−0.740	to	0.341
Reducing the amount of work	0.033	0.675	−0.200	to	0.308
R^2^	0.180				
Total Adjusted R^2^	0.092				

(^a^) Activities of daily living, (^b^) Instrumental activities of daily living. (^c^) In-home activities: reading books, listening to music, drawing, exercises, horticulture, handicrafts. (^d^) Out-of- home personal leisure activities: walking, eating out, drinking coffee, and shopping. (^e^) Out-of-home group activities: neighborhood associations, hobby associations, and family caregiver associations. (^f^) Cultural and entertainment out-of-home activities: sports, travel, watching movies, and watching art.

**Table 5:** Multiple regression analysis results with CES-D as the dependent variable (*n* = 171).

Variables	Coefficient β	*p*-Value	95% Cl		
Demographic characteristics					
Gender (female = 1, male = 0)	0.110	0.190	−0.613	to	3.056
Age	0.041	0.625	−0.050	to	0.083
Household economic situation (1 = Very poor to 5 = Very rich)	−0.189	0.019	−1.446	to	0.129
Care Recipients’ Situation					
ADL care provided ^(a)^	0.128	0.215	−0.120	to	0.531
IADL care provided ^(b)^	−0.181	0.069	−0.684	to	0.026
Cognitive impairment	0.110	0.190	−0.613	to	3.056
Number of secondary caregivers	−0.022	0.795	−0.352	to	0.270
Experience of caregiver giving up leisure activity (Have quit = 1, Never = 0)					
In-home activities ^(c)^	−0.085	0.326	−2.835	to	0.949
Out-of-home personal activities ^(d)^	0.136	0.128	−0.361	to	2.841
Out-of-home group activities ^(e)^	0.176	0.042	0.059	to	3.337
Cultural and entertainment out-of-home activities ^(f)^	0.047	0.593	−1.135	to	1.977
Experience of caregiver changing work situation (Have quit = 1, Never = 0)					
Quitting jobs	0.053	0.524	−1.108	to	2.164
Changing jobs	0.081	0.432	−2.732	to	6.349
Taking care leave (long absence from work)	0.217	0.023	0.940	to	12.66
Changing the employment condition (ex. From full time to part time job)	−0.258	0.023	−9.359	to	0.711
Reducing the amount of work	−0.016	0.858	−2.291	to	1.911
R^2^	0.450				
Total Adjusted R^2^	0.203				

(^a^) Activities of daily living, (^b^) Instrumental activities of daily living. (^c^) In-home activities: reading books, listening to music, drawing, exercises, horticulture, handicrafts. (^d^) Out-of- home personal leisure activities: walking, eating out, drinking coffee, and shopping. (^e^) Out-of-home group activities: neighborhood associations, hobby associations, and family caregiver associations. (^f^) Cultural and entertainment out-of-home activities: sports, travel, watching movies, and watching art.

## Data Availability

The data in this study is sensitive, and in the surveyed nurse-stations, disclosure of raw data is not allowed even if anonymous.
